# Supplementary material for: Trends of Korean Medicine Treatment for Parkinson’s Disease in South Korea: A Cross-Sectional Analysis Using the Health Insurance Review and Assessment Service–National Patient Sample Database
Source: Healthcare (Basel). 2025 May 21;13(10):1207. doi: 10.3390/healthcare13101207 (PMC12111431; doi:10.3390/healthcare13101207)
Supplement: Supplementary file 1 [file healthcare-13-01207-s001.zip › healthcare-3550643-supplementary.pdf]

Table S1. Yearly statistics for the total number of patients diagnosed with PD and the number of KM and non-KM users

Year	Total	Non-KM users		KM users	
	N	N	%	N	%
2010	1,333	1,248	93.6	85	6.4
2011	1,451	1,338	92.2	113	7.8
2012	1,605	1,465	91.3	140	8.7
2013	1,751	1,587	90.6	164	9.4
2014	1,901	1,701	89.5	200	10.5
2015	1,901	1,713	90.1	188	9.9
2016	2,033	1,831	90.1	202	9.9
2017	2,079	1,844	88.7	235	11.3
2018	2,227	1,996	89.6	231	10.4
2019	2,260	2,021	89.4	239	10.6

KM: Korean medicine, PD: Parkinson's disease

Table S2. Number of bills and medical costs per service category for NHI-covered KM services

Category	Bills		Total cost	
	No. of claims (cases)	Percentage (%)	Total cost ($)	Percentage (%)
Acupuncture	18,806	28.8	376,008	50.6
Consultation	18,753	28.8	138,398	18.6
Physical therapy	8,094	12.4	8,744	1.2
Moxibustion	6,326	9.7	54,460	7.3
Cupping	6,279	9.6	67,359	9.1
Electroacupuncture	4,666	7.2	31,376	4.2
Dispensing	1,834	2.8	1,059	0.1
Meal service fee	111	0.2	14,383	1.9
Admission	110	0.2	47,911	6.5
Rehabilitation assistive devices	102	0.2	463	0.1
Chuna therapy	50	0.1	1,555	0.2
Examination	47	0.1	202	0.0
Others	15	0.0	336	0.0
Procedure	10	0.0	394	0.1
KM psychotherapy	3	0.0	30	0.0

KM: Korean medicine, NHI: National Health Insurance

Table S3. Number of bills and medical costs for frequently used KM services

Code (fee for service)	Service	Bills		Cost	
		No. of claims (cases)	Percentage (%)	Total cost ($)	Percentage (%)
40012	Acupuncture (two regions)	14,703	21.7	177,227	32.2
40080	Acupuncture (perforating)	11,728	17.3	143,606	26.1
40701	Transcutaneous infrared irradiation therapy	7,184	10.6	7,393	1.3
40091	Electroacupuncture	4,666	6.9	31,376	5.7
40060	Intra-articular acupuncture	4,091	6.0	25,095	4.6
40321	Dry cupping therapy	3,869	5.7	45,951	8.3
40011	Acupuncture (one region)	3,786	5.6	14,550	2.6
40306	Moxibustion (indirect moxa cone)	3,578	5.3	30,786	5.6
40400	Pattern identification technique	3,182	4.7	9,485	1.7
40307	Moxibustion (indirect instrument)	1,763	2.6	11,062	2.0

KM: Korean medicine

Table S4. Primary diagnoses in claims for PD-related KM services based on the first three digits

Rank	Diagnosis code	Diagnosis	No. of claims (cases)	Percentage (%)
1	M54	Dorsalgia	37,478	28.1
2	M79	Other and unspecified soft tissue disorders, not elsewhere classified	11,124	8.3
3	M17	Osteoarthritis of knee	6,954	5.2
4	M75	Shoulder lesions	5,441	4.1
5	S33	Dislocation and sprain of joints and ligaments of lumbar spine and pelvis	4,728	3.5
6	M25	Other joint disorder, not elsewhere classified	4,691	3.5
7	M62	Other disorders of muscle	4,570	3.4
8	U23	Head wind	4,346	3.3
9	R25	Abnormal involuntary movements	3,595	2.7
10	U**	(Blinded)	3,391	2.5
11	U24	Numbness	2,487	1.9
12	U30	Moving impediment	1,922	1.4
13	K30	Functional dyspepsia	1,859	1.4
14	M48	Other spondylopathies	1,850	1.4
15	G51	Facial nerve disorders	1,717	1.3
16	I69	Sequelae of cerebrovascular disease	1,648	1.2
17	R42	Dizziness and giddiness	1,627	1.2
18	S93	Dislocation and sprain of joints and ligaments at ankle, foot and toe level	1,304	1.0
19	M51	Other intervertebral disc disorders	1,204	0.9
20	S43	Dislocation and sprain of joints and ligaments of shoulder girdle	1,149	0.9

KM: Korean medicine, PD: Parkinson’s disease

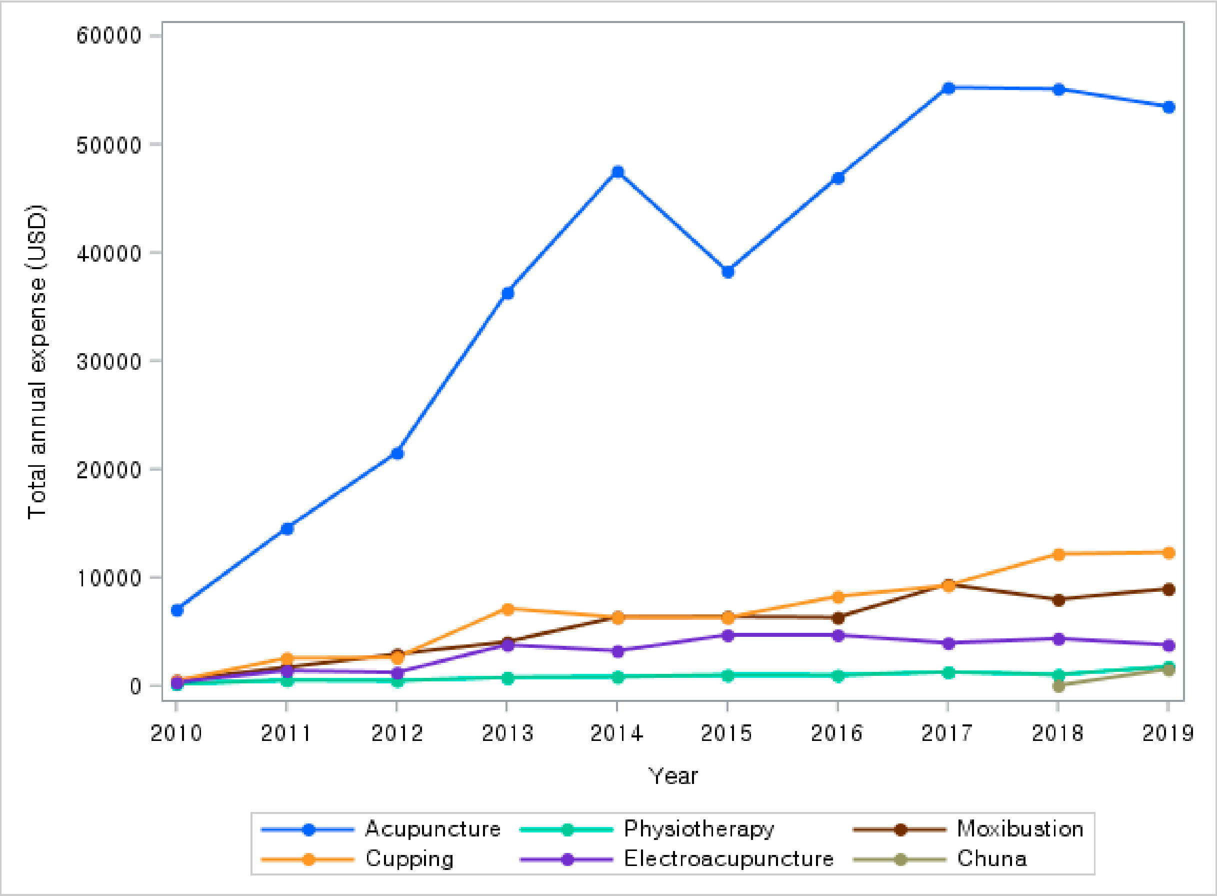

Figure S1. Trends in total annual expenses for major treatment modalities of KM services. KM: Korean medicine
